# Benefits of condylar distraction in patients with temporomandibular dysfunction

**DOI:** 10.1080/07853890.2021.1896442

**Published:** 2021-09-28

**Authors:** Rita Castro, Pedro Lima, Marion Fernandes, João Martinho, Paula Moleirinho Alves, Ângela Maria Pereira

**Affiliations:** a Department of Physiotherapy, Escola Superior de Saúde Egas Moniz (ESSEM), Egas Moniz Cooperativa de Ensino Superior, Caparica, Portugal; b Centro de Investigação Interdisciplinar Egas Moniz (CiiEM), Egas Moniz Cooperativa de Ensino Superior, Caparica, Portugal; c Hospital Garcia de Orta, Almada, Portugal

## Abstract

**Introduction:**

Temporomandibular disorders (TMD) are considered as a heterogeneous group of psychophysiological disorders of the stomatognathic system [1]. They are frequently initiated by pain, joint sounds and limited function/mandibular movement, being considered one of the main cause of orofacial pain of non-dental origin [2]. Among the TMD of articular origin, disc displacements with and without reduction, osteoarthrosis and osteoarthritis are the most frequent alterations in patients [3]. Conservative and non-invasive treatment is considered as the first choice [4] and physical therapy is indicated as one of the most frequently recommended types of treatment [5]. The objective of the present study is to analyse the effects of the condylar distraction technique after four weeks of intervention regarding pain, joint noises and amplitude of mouth opening.

**Materials and methods:**

A prospective, quasi-experimental study, was performed. We include 16 patients with a diagnosis of temporomandibular joint dysfunction according to the Research Diagnostic Criteria for temporomandibular disorder. Patients were randomised into two groups: 8 (39.0 ± 13.2 years old) in the experimental group (EG) and 8 (39.8 ± 13.9 years old) in the control group (CG). Patients from both groups performed home-based exercises daily at home, GE patients performed 4 sessions of physiotherapy, having undergone condylar distraction techniques, CG patients did not perform any other type of intervention. All patients were evaluated before (T0) and after (T1) the intervention. The range of the mandible was evaluated through a digital calliper, the intensity of the pain through the numeric scale of the pain and the presence of articular noises, through palpation. All participants signed informed consent. The study was approved by the Ethics Committee of the Egas Moniz

**Results:**

Condylar distraction technique increased motion range values of the mandible from T0 to T1 in GE group (*p* = .012) and decrease value of pain intensity in T1 when compared to T0 in the GE group (*p* = .008). The obtained results were analized using Student’s *t* test. There were no changes in joint noises when comparing T0 with T1 in both groups.

**Discussion and conclusions:**

It is concluded that the condylar distraction technique has positive effects on pain and range of motion of the mandible. However, joint noises remained present after intervention, concluding that condylar distraction has no effect on noise reduction. Recent evidence suggests that manual therapy is a legitimate treatment for TMD promoting improvement in mouth opening and reduction in jaw pain [6]. However, further investigations should be carried out with larger samples in the future.
